# High-dose vitamin C on sepsis: Protocol of a prospective, multi-centered, double-blinded, randomized, and placebo-controlled superiority study

**DOI:** 10.3389/fmed.2022.950246

**Published:** 2022-09-15

**Authors:** Bing Zhao, Mengjiao Li, Wenwu Sun, Jian Li, Leshan Liu, Yihui Wang, Silei Sun, Lili Xu, Xing Qi, Mengqi Xie, Yuhua Zhou, Tongtian Ni, Yi Yao, Peili Chen, Meiling Yu, Weisong Jiang, Ning Ning, Huiqiu Sheng, Erzhen Chen, Ruilan Wang, Chaoyang Tong, Yu Cao, Mingwei Sun, Enqiang Mao

**Affiliations:** ^1^Department of Emergency of Ruijin Hospital, Shanghai Jiao Tong University School of Medicine, Shanghai, China; ^2^Department of The Clinical Research, Ruijin Hospital Affiliated to Shanghai Jiao Tong University School of Medicine, Shanghai, China; ^3^Department of Critical Care, First People’s Hospital Affiliated to Shanghai Jiao Tong University School of Medicine, Shanghai, China; ^4^Department of Emergency, Zhongshan Hospital Affiliated to Fudan University, Shanghai, China; ^5^Department of Emergency, West China Hospital, Sichuan University, Sichuan, China; ^6^Department of Emergency, Sichuan Provincial People’s Hospital, Sichuan, China

**Keywords:** high dose, vitaminc, sepsis, antioxidant, clinical trial

## Abstract

**Background:**

Sepsis is an inflammatory syndrome with life-threatening organ dysfunction and high mortality. In the recent 10 years, high-dose intravenous injection of vitamin C, the first-line antioxidant of humans, has received highlighted attention in the field of critical care. The study aims to examine the efficacy and safety of high-dose intravenous injection of vitamin C in the treatment of sepsis.

**Methods and design:**

Here, we are conducting a prospective, multi-centered, double-blinded, randomized, and placebo-controlled superiority study named High-Dose Vitamin C on Sepsis (HDVCOS). A total of 620 participants diagnosed with sepsis in four participating sites across China that satisfy the eligibility criteria will be randomized at a ratio of 1:1 to receive treatment with a high-dose intravenous injection of vitamin C (200 mg/kg/24 h) or placebo (saline) for 4 days. The primary outcome is 28 days of mortality. The secondary outcomes include the incidence of organ failure, Sequential Organ Failure Assessment (SOFA) score change, organ support, the relationship between plasma vitamin C concentration and outcomes, and adverse events.

**Conclusion:**

The findings of this study will provide potential evidence for high-dose intravenous injection of vitamin C in the treatment of sepsis.

**Clinical trial registration:**

[http://www.chictr.org.cn/showprojen.aspx?proj=29851], identifier [ChiCTR1800017633].

## Introduction

Sepsis is defined as an inflammatory syndrome with life-threatening organ dysfunction resulting from a dysregulated host response to infection (1). The global annual incidence of sepsis is up to 31 million cases, including 19.4 million cases of severe sepsis (2). The incidence of sepsis in China is 461 per 100,000 population (3), according to the latest data. The management of sepsis, including antibiotics, source control, fluid resuscitation, vasopressors, and organ support, has improved a lot in the last 30 years, but sepsis-related mortality remains high, with 74 deaths per 100, 000 population worldwide (2) and 66.7 deaths per 100,000 population in China (4). New interventions to improve mortality need to be explored and developed.

After the onset of sepsis, uncontrolled oxidative stress-induced systemic inflammatory response syndrome (SIRS) contributes to progressive organ dysfunction and death (5). In the recent 10 years, a high-dose intravenous injection of vitamin C, the first-line antioxidant of humans, has received highlighted attention in the field of critical care (6). Its beneficial effect in critical illness has been demonstrated by several small-scale clinical trials and animal studies, such as severe burn (7), severe acute pancreatitis (8), ischemic reperfusion injury (9–11), and sepsis (12). Still, the effect of a high-dose intravenous injection of vitamin C on sepsis remains controversial according to the recent retrospective work of the cohort study (13) and the real-world study (14). In the past several randomized control trials, high-dose vitamin C was shown to fail to improve organ function scores and inflammatory biomarkers but to improve the survival rate in sepsis by the results of the CITRIS-ALI study (*N* = 167) (15). However, David et al. failed to demonstrate that intravenous vitamin C therapy could significantly improve the survival rate in patients with sepsis (*N* = 124) (16). Therefore, a larger trial exploring the effect of high-dose intravenous vitamin C on sepsis seems necessary. Therefore, we have designed this prospective, multi-centered, double-blind, randomized, placebo-controlled study to explore the effect of high-dose vitamin C on sepsis.

## Methods and analysis

### Study design

We have designed the High-Dose Vitamin C on Sepsis (HDVCOS) study as a prospective, multi-centered, double-blinded, randomized, placebo-controlled, superior clinical trial. Till now, five centers in China have been involved. The trial protocol is approved by the Clinical Trail Ethics Committee of Ruijin Hospital of Shanghai Jiao Tong University School of Medicine (2018-145) and all the participating centers. This trial has been registered in the Chinese Clinical Trial Registry (ChiCTR1800017633). A total of 620 participants diagnosed with sepsis that satisfy the eligibility criteria will be randomized at a ratio of 1:1 to receive treatment with a high-dose intravenous injection of vitamin C (200 mg/kg/24 h) or placebo (saline) for 4 days.

### Study population

**FIGURE 1:**
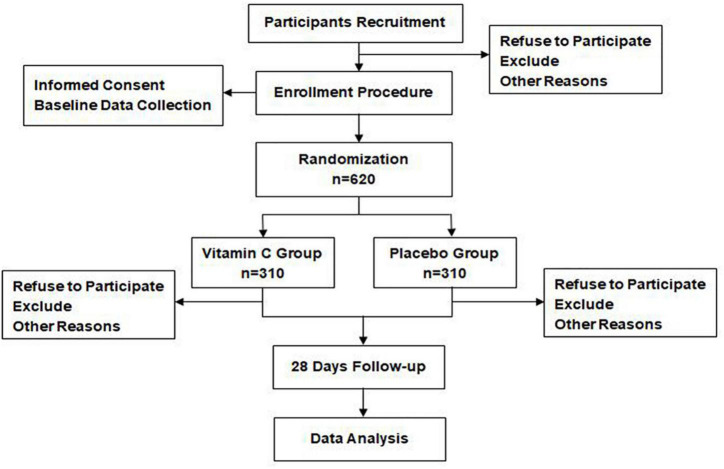
Participants flow chart.

Any patient admitted to the participating sites and diagnosed with sepsis according to sepsis 3 definition (1) will be considered for enrollment. Figure 1 shows the flow chart throughout the study. The recruitment was initiated in August 2019.

#### Inclusion criteria

The inclusion criteria were as follows:

1. Patients aged above 18 years.
2. Patients meeting the diagnostic criteria of sepsis, which is suspected infection and organ dysfunction (total Sequential Organ Failure Assessment (SOFA) score ≥ 2 points).
3. Patients within 72 h from the establishment of sepsis diagnosis to inclusion.
4. Patients who avoided pregnancy during the whole study period.
5. Patients who were able to read and write in Chinese and should provide informed consent.

#### Exclusion criteria

The exclusion criteria were as follows:

1. Patients or their surrogate refuse to receive cardiovascular (vasopressor agent), respiratory (mechanical ventilation), or renal (renal replacement therapy) supports during the treatment period.
2. Pregnant patients or in lactation.
3. Patients with poorly controlled chronic organ failure, defined as: (1) chronic cardiovascular dysfunction requiring long-term mechanical hemodynamic support or inotropes support, (2) chronic obstructive pulmonary disease requiring home oxygen, (3) chronic hepatic dysfunction classified as Child-Pugh C, (4) chronic renal disease with an eGFR < 60 ml/min/1.73 m^2^ or serum creatinine >150 μmol/L.
4. Patients allergic to vitamin C.
5. Patients who are participating or have participated in other investigational studies.
6. Patients who weigh more than 100 kg.
7. Patients with kidney stone.

### Randomization, Blinding, and allocation

Participants are randomly assigned to the vitamin C or normal saline (NS) groups in a 1:1 ratio. The randomization sequence is generated with SAS v. 9.2 (SAS Institute Inc., United States) using stratified block randomization with a block length of 10 and stratified by site. A randomized envelope is made by a statistician in our clinical research center. The sequence number is marked outside the envelope and the medication puzzled for vitamin C or NS is sealed inside. The randomization information is blinded to all the participants in the trials except the unblinding drug preparing nurse (UDPN).

After consent is obtained and the inclusion/exclusion criteria are verified, the principal investigator (PI) in each participating site (PS) hands the unique randomized envelope of the participant to UDPN. The UDPN unseals the envelope and disposes the vitamin C or NS into an opaque 50 ml syringe according to grouping information inside the envelope. UDPN gives the opaque syringe to the therapy nurse and is independent of the following medical process. The outcome assessor and statistician are blinded to randomization and are not involved in treatment procedures.

### Medication and intervention

Patients receive the first dose of vitamin C or NS within 2 h after randomization.

#### Regular treatment

The sepsis 3.0 guideline will be followed for the regular management of patients with sepsis (17). This includes fluid resuscitation, antibiotics, vasopressor titration, mechanical ventilation and ventilator weaning strategies, blood transfusion, nutrition, renal replacement therapy, delirium management, and glycemic control.

Group 1: Vitamin C group

The vitamin C (Brilliant, China) with a dose of 200 mg/kg/24 h is given by continuous injection through the central venous with a speed of 5 ml/h for 4 days.

Group 2: NS group

The.9% NS (Baxter, China) with the same volume as corresponding vitamin C is given by continuous injection through the central venous with a speed of 5 ml/h for 4 days.

### Outcomes

#### Primary outcome

The primary aim of this study is to prospectively assess the effect of high-dose vitamin C therapy on 28 days of mortality in patients with sepsis.

#### Secondary outcomes

The secondary outcomes are as follows:

1. The incidence of organ failure within 28 days: organ failure is defined as at least three points on the SOFA score assigned in a particular organ system (cardiovascular, hepatic, respiratory, renal, and coagulation system) (18).
2. Change in the SOFA score on days 1, 3, 7,14, and 28.
3. The duration of organ support within 28 days: Organ support is defined as mechanical ventilation, renal replacement therapy, and vasoactive drug application.
4. Plasma concentration of vitamin C on days 0, 3, 4, and 7 and its relationship with outcomes and adverse events
5. The change of inflammatory indicators (C-reactive protein and procalcitonin) on days 1, 3, 7, 14, and 28.

### Adverse events

An adverse event (AE) is “any untoward medical occurrence in a human subject, including any abnormal sign (e.g., abnormal physical exam or laboratory finding), symptom, or disease” and occurs during a subject’s participation in research.

AEs include the following:

1. crystal in urine
2. respiratory failure
3. thromboembolic disease
4. arrhythmias
5. delirium
6. anemia
7. coagulopathy

Severe AEs (SAEs) include the following:

1. unexplained acute kidney failure
2. life-threatening events
3. results in prolongation of the existing hospitalization
4. persistent or significant disability/incapacity
5. death

Both AEs and SAEs were evaluated at 8:00 am every day. They will be recorded and reported to the PI of each research site and evaluated if they are related to this study. Once confirmed, the given medicine should be discontinued immediately, and the patients should be treated appropriately. Both AEs and SAEs will be reported.

### Sample size

The sample size calculation is based on the primary outcome of 28 days of mortality in patients with sepsis. It was estimated that the sepsis mortality rate within 28 days would be 40%. We used the superiority test for two proportions based on the one-sided 2.5% of type I error probability and 80% of power. A total of 620 participants are required to detect a decrease of 10% mortality rate and 10% dropout rate. The sample size was calculated by PASS 15 software (NCSS, Kaysville, UT, United States).

### Data collection

All data are collected using a web-based database in each participating center (Table 1). When participants are withdrawn from the study, they are not replaced. Patients that are lost during the study period will be incorporated into the analysis until their final lost date. Missing data are replaced by chained equations using multiple imputations if these missing data are judged to be random.

**TABLE 1:** Collected data and outcomes.

Procedures	Day 0	Day 1	Day 3	Day 4	Day 7	Day 14	Day 28
Eligibility criteria	X						
Informed consent	X						
Demographic and medical data	X						
Vital signs	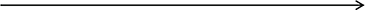						
Fluid balance	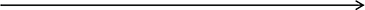						
Routine laboratory test*	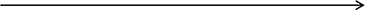						
Inflammatory indicators #	X	X	X	X	X	X	X
Plasma vitamin C concentration	X		X	X	X		
SOFA score	X	X	X	X	X	X	X
APACHEII score	X						
Organs support	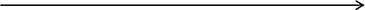						
Vasoactive drugs	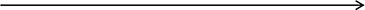						
Adverse events	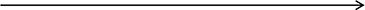						
Death							X
Hospital stay							X

*Routine laboratory tests include the following: white blood cell (WBC) count, hemoglobin, thrombocytes, blood gas analysis, electrolytes, urea, creatinine, aspartate transaminase, alanine transaminase, lactate dehydrogenase, alkaline phosphatase, NT-proBNP, TnI, and coagulation test. #Inflammatory indicators include the following: C-reactive protein (CRP) and procalcitonin (PCT). SOFA, Sequential Organ Failure Assessment; APACHEII, Acute Physiology And Chronic Health Evaluation II.

### Statistical analysis

Categorical data will be described with frequency or ratio. Continuous variables will be described using mean and SD for normally distributed variables or median and interquartile range (IQR) for non-normally distributed variables. Categorical variables will be compared using the chi-square test or the Fisher exact test. Continuous variables will be compared using the t-test for normally distributed variables or the Wilcoxon rank-sum test for non-normally distributed variables. The efficacy analysis will be based on the intention-to-treat principle and compared with the per-protocol principle subset.

The primary outcome of 28 days mortality will be compared between groups using the chi-square test or the Fisher exact test. Superiority could be calculated if the upper bound of 28 days of mortality was lower than the 2.5% superiority margin. Logistic analysis with a 95% CI is used to calculate the odds ratio of each risk factor.

The secondary outcomes of the SOFA score change and inflammatory indicators change on days 1, 3, 7, 14, and day 28 between two groups will be analyzed using a generalized linear model. The incidence of organ failure and adverse events occurrence will be compared using the chi-square tests or the Wilcoxon rank-sum test. The duration of organ support and vitamin C concentration on days 0, 3, 4, and day 7 will be compared using the *t*-test or the Wilcoxon rank-sum test. All these analyses are performed two-sided at the 5% significance level. SAS v. 9.2 (SAS Institute Inc., United States) is used for the analysis.

### Monitoring

A trial management committee comprising of the principal investigators, supported by the co-investigators from the other participating sites, was formed. The trial management committee is responsible for the day-to-day running and management of the trial. In order to standardize the research procedures, the researchers, physicians, and nurses are trained in standard clinical practice. A standard case report form is developed for every participant. Obtained data is stored strictly. Data will be analyzed by a statistician after the removal of any participant identifier information. Data are analyzed at the completion of this study.

## Discussion

In the present study, we would conduct a prospective, multi-centered, double-blinded, randomized, placebo-controlled superiority clinical trial named HDVCOS. Its aim is to testify to the efficacy and safety of high-dose vitamin C in the treatment of sepsis.

The clinical clue of the supplement of vitamin C in critical illnesses (such as trauma, sepsis, and hemorrhagic shock) has been confirmed for a long time. Severe vitamin C deficiency (plasma concentrations < 11 μmol/L) is commonly seen in nearly 40% patients with sepsis (19). Vitamin C deficiency was reported to be closely related to poor prognosis (20). The reason may be that vitamin C exerts many important functions in our physiological activity: (1) directly erasing reactive oxidative species (ROS); (2) acting as a key cofactor for the biosynthesis of endogenous catecholamines, vasopressin, and cortisol; and (3) functioning as an immune enhancer (21). In recent years, even though two clinical trials separately investigate the effect of intravenous vitamin C on sepsis (15, 16), the conclusion is still elusive. The reason may be the relatively low participants number (*N* = 167 and *N* = 124, separately) and the undefined optimal vitamin C dose and plasma level. Recently, the results of a large multi-center randomized trial were just published and the results indicated no benefit of vitamin C in adults with sepsis (22). However, Asian people were not included in this study. Moreover, the time interval between established diagnosis of sepsis and the application of vitamin C was not provided, which is important to evaluate the effect of vitamin C. Finally, the plasma vitamin C level during and after infusion in this study was not indicated. It was not sure whether the plasma vitamin C concentration was enough for a septic adult. None of the studies to date have obtained this important information either. In our study, plasma vitamin C concentration is measured continuously within the first 7 days, and the outcome will provide important information on this aspect.

In this trial, we will give the vitamin C in a way of sustained injection (200 mg/kg/24 h, 1 g/h) for 4 days. Other published studies (12, 15, 22) used the same dosage of vitamin C (200 mg/kg/24 h) for 4 days in total, but they gave vitamin C in a bolus injection way. According to the study by De Grooth et al. (23), a sustained injection of 10-g vitamin C for 48 h led to a sustained level of vitamin C approximately five times above the normal level, which might be more helpful to prevent hypovitaminosis than bolus injection. In our study, we will test the plasma vitamin C concentration with high-performance liquid chromatography on days 0, 3, 4, and 7, and the result may provide information on optimal drug delivery ways and plasma concentrations.

The most concern of high-dose vitamin C safety is its potential harm to renal function and the formation of kidney stones (24). A previous case report showed that high-dose oral intake of vitamin C for 6 years led to high urine oxalate excretion and ureteral stone formation in a 9-year-old child. However, there was no reported case of renal stone as an adverse event of short-term intravenous usage of high-dose vitamin C (25). Furthermore, no adverse event was reported in critical illness clinical trials. Anyway, the renal function change of enrolled patients should receive timely evaluation. Patients with a history of renal oxalate stones will not be enrolled in the study.

There are some limitations to this study. First, the rapid degradation quality of vitamin C and the inconvenient availability of high-performance liquid chromatography could influence the accuracy of plasma vitamin C concentration detection. Second, patients with kidney stones were excluded from the trial, which may exclude most of the patients with sepsis originating from the urinary tract.

Vitamin C is a cheap and easily available agent. We expect that the results of this study will provide a scientific rationale for the use of high-dose vitamin C in improving the prognosis of patients with sepsis.

## Trial status

The trial start date was 1 August 2019. Considering the influence of COVID-19, the anticipated recruitment end date will be 31 December 2023.

## Ethics statement

The studies involving human participants were reviewed and approved by Clinical Trail Ethics Committee of Ruijin Hospital of Shanghai Jiao Tong University School of Medicine. The patients/participants provided their written informed consent to participate in this study.

## Author contributions

EM was the principal of this clinical trial. BZ, JL, YW, RW, CT, YC, HS, MS, and EC conducted and coordinated the process. LL designed the online database. SS and LX collected the simples. XQ, MX, YZ, TN, YY, PC, MY, and WJ were responsible for collecting the data. BZ, ML, and WS wrote the draft. RW, CT, YC, MS, and MQ were the primary investigator of five centers. All authors contribute to the refinement of the study protocol and have read and approved the final version.
